# Supplementary material for: Identification of photoperiod-induced specific miRNAs in the adrenal glands of Sunite sheep (Ovis aries)
Source: Front Vet Sci. 2022 Jul 22;9:888207. doi: 10.3389/fvets.2022.888207 (PMC9354845; doi:10.3389/fvets.2022.888207)
Supplement: Supplementary file 1 [file Data_Sheet_1.ZIP › Supplementary file/miRDeep2 file.zip!/miRDeep2 file/Know_Structure/LP42A1/oar-mir-200a.pdf]

miRBase precursor : oar-mir-200a
Total read count : 143
oar-miR-200a read count : 139
remaining reads : 4

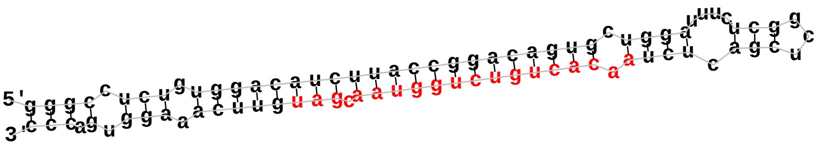

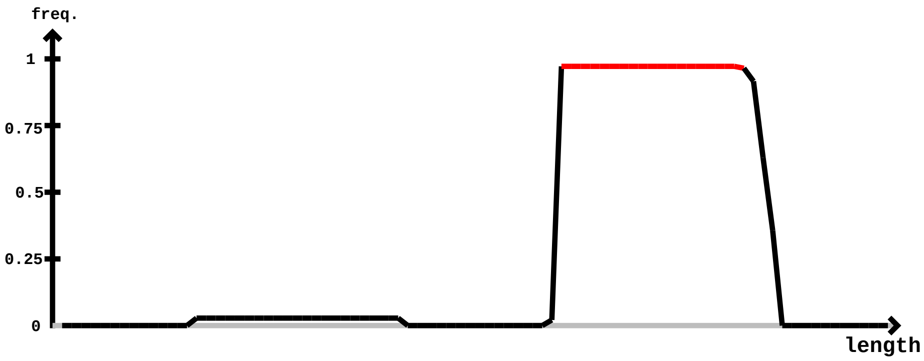

oar-miR-200a

```
5'- gggccucuguggacaucuuaccggacagugcuggauuucucggcucgacucuaacacugucugguaacgauguucaaaggugaccc   -3'    exp
    ((((.(((.(((((((((((((((((((((.((((....(((...))).)))).))))))))))))).)))))))).))).).)))    reads      mm         sample
    .............caucuuaccggacagugcugga...................................................    4          0          seq
    ..................................................cuaacacugucugguaaAgaug..............    3          1          seq
    ...................................................uaacacugucugguaaAga................    1          1          seq
    ...................................................uaacacugucugguaacgau...............    2          0          seq
    ...................................................uaacacugucugguaaAgau...............    5          1          seq
    ...................................................uaacacugucugguaaAgaug..............    17         1          seq
    ...................................................uaacacugucugguaacgaug..............    21         0          seq
    ...................................................uaacacugucugguaacgaugu.............    39         0          seq
    ...................................................uaacacugucugguaacgauguu............    51         0          seq
```
